# Different responses of skeletal muscles to femoral artery ligation-induced ischemia identified in BABL/c and C57BL/6 mice

**DOI:** 10.3389/fphys.2022.1014744

**Published:** 2022-09-16

**Authors:** Huiyin Tu, Junliang Qian, Dongze Zhang, Aaron N. Barksdale, Michael C. Wadman, Iraklis I. Pipinos, Yu-Long Li

**Affiliations:** ^1^ Department of Emergency Medicine, University of Nebraska Medical Center, Omaha, NE, United States; ^2^ Department of Surgery, University of Nebraska Medical Center, Omaha, NE, United States; ^3^ Department of Cellular and Integrative Physiology, University of Nebraska Medical Center, Omaha, NE, United States

**Keywords:** animal model, femoral artery ligation, injury, ischemia, limb, neuromuscular junction, peripheral arterial disease, skeletal muscle

## Abstract

Peripheral arterial disease (PAD) is a common circulatory problem in lower extremities, and the murine ischemic model is used to reproduce human PAD. To compare strain differences of skeletal muscle responses to ischemia, the left femoral artery was blocked by ligation to reduce blood flow to the limb of BALB/c and C57BL/6 mice. After 6 weeks of the femoral artery ligation, the functional and morphological changes of the gastrocnemius muscle were evaluated. BALB/c mice displayed serious muscular dystrophy, including smaller myofibers (524.3 ± 66 µM^2^), accumulation of adipose-liked tissue (17.8 ± 0.9%), and fibrosis (6.0 ± 0.5%), compared to C57BL/6 mice (1,328.3 ± 76.3 µM^2^, 0.27 ± 0.09%, and 1.56 ± 0.06%, respectively; *p* < 0.05). About neuromuscular junctions (NMJs) in the gastrocnemius muscle, 6 weeks of the femoral artery ligation induced more damage in BALB/c mice than that in C57BL/6 mice, demonstrated by the fragment number of nicotinic acetylcholine receptor (nAChR) clusters (8.8 ± 1.3 in BALB/c vs. 2.5 ± 0.7 in C57BL/6 mice, *p* < 0.05) and amplitude of sciatic nerve stimulated-endplate potentials (EPPs) (9.29 ± 1.34 mV in BALB/c vs. 20.28 ± 1.42 mV in C57BL/6 mice, *p* < 0.05). More importantly, 6 weeks of the femoral artery ligation significantly weakened sciatic nerve-stimulated skeletal muscle contraction in BALB/c mice, whereas it didn’t alter the skeletal muscle contraction in C57BL/6 mice. These results suggest that the femoral artery ligation in BALB/c mice is a useful animal model to develop new therapeutic approaches to improve limb structure and function in PAD, although the mechanisms about strain differences of skeletal muscle responses to ischemia are unclear.

## Introduction

Peripheral arterial disease (PAD) is a circulatory problem causing a reduced blood flow through the arteries, most commonly in the lower extremities and confers a high risk of cardiovascular events and death (Criqui and Aboyans 2015). PAD, as prevalent as coronary artery disease, is the third leading cause of atherosclerotic cardiovascular morbidity, following coronary artery disease and stroke and increased by 28.7% in low-income and middle-income countries and by 13.1% in high-income countries between the years 2000 and 2010, affects >200 million individuals worldwide (Fowkes et al., 2013; Criqui and Aboyans 2015). The most common presentation of PAD is either intermittent claudication (pain during walking that is relieved with rest) or critical limb ischemia (CLI, pain at rest with or without tissue necrosis or gangrene) (Beckman et al., 2019). Patients with intermittent claudication have a combined annual amputation and mortality rate of 2%–4% per year, whereas patients with CLI have a 6-months amputation risk of 25%–40% and an annual mortality rate as high as 20% (Juergens, Barker, and Hines 1960; Da Silva et al., 1979; Ouriel 2001).

Preclinical rodent models of hindlimb ischemia have been used for decades to simulate PAD pathology (Couffinhal et al., 1998; Dokun et al., 2008; Okeke and Dokun 2018). Most current pre-clinical models are designed to produce acute ischemia which leads to muscle necrosis and inflammation (Krishna, Omer, and Golledge 2016). PAD patients, however, most commonly present with chronic ischemia suggesting that more representative models are needed to evaluate therapeutic modalities that can be potentially translated to clinical practice (Krishna, Omer, and Golledge 2016).

Both C57BL/6 (Pipinos et al., 2008; Mohiuddin et al., 2019) and BALB/c (Roseguini et al., 2015; Goldberg et al., 2019) mice were used to induce pre-clinical PAD models by femoral artery ligation. However, they showed strain-dependent variations in acute ischemic muscle injury (Dokun et al., 2008; McClung et al., 2016; Schmidt et al., 2018) and hindlimb collateral artery development (Helisch et al., 2006; Chalothorn et al., 2007; Chalothorn and Faber 2010). This variability in the strains of rodents possibly slows down the translation from basic research to clinical practice (Krishna, Omer, and Golledge 2016). In this study, we compared the morphological and functional alterations in gastrocnemius muscle between C57BL/6 and BALB/c mice after their femoral artery ligation and found that BALB/c mice pathologically and functionally simulated the clinical symptom of PAD.

## Materials and methods

### Animal protocols and operative procedures for induction of hindlimb ischemia

All experimental procedures were approved by the University of Nebraska Medical Center Institutional Animal Care and Use Committee (approved IACUC number: 16-140-01-FC) and were carried out in accordance with the National Institutes of Health (NIH Publication No. 85-23, revised 1996) and the American Physiological Society’s “Guides for the Care and Use of Laboratory Animals”. The mice were housed under controlled temperature and humidity with a 12:12-h dark–light cycle and provided water and mouse chow *ad libitum*. For all invasive procedures, buprenorphine SR-LAB (1.0 mg/kg, s. c., ZooPharm, LLC, Laramie, WY, United States) served as an analgesic to provide 3 days of analgesia for post-operation. After *in vivo* experiments were performed, mice were euthanized with 0.52 ml/kg of Fatal-Plus euthanasia solution (about 200 mg/kg pentobarbital, i. p., Vortech Pharmaceuticals, Dearborn, MI, United States).

A total of 63 male mice (12–14 weeks of age, 31 C57BL/6 and 32 BALB/c mice) weighing 23–27 g (Charles River Laboratory, Wilmington, MA; and Jackson Laboratory, Bar Harbor, ME) were randomly assigned into 4 groups: C57BL/6 sham, C57BL/6 femoral artery ligation (C57BL/6 Isch), BALB/c sham and BALB/c femoral artery ligation (BALB/c Isch). Under anesthesia with 2% isoflurane inhalant, the left femoral artery of the mouse was exposed through a longitudinal incision on the anterior thigh of the left hindlimb, and ligated proximal to the superficial epigastric artery with 7-0 silk ligature. Sham-operated animals underwent the same protocol except for no femoral artery ligation. The level of anesthesia was continuously monitored by testing the respiratory patterns and toe pinch reflex. A heating pad (ATC 2000; WPI) was used to maintain body temperature at 37°C until the animal woke up. The skin was closed with ethilon 4-0 monofilament non-absorbable suture, and the suture was removed after 10–14 days. Final experiments, including measurements of gastrocnemius muscle contraction, endplate potentials (EPPs), and muscle and neuromuscular junction (NMJ) morphologies were performed at 6 weeks after the femoral artery ligation.

### *In-situ* recording of the gastrocnemius muscle contraction and endplate potentials

The gastrocnemius muscle contractile force and EPPs were recorded as described previously. Under anesthesia (800 mg/kg urethane and 40 mg/kg chloralose, i. p.), the mouse was kept in prone position and maintained at 37°C, the middle and distal ends of the left gastrocnemius muscle were isolated and moistened by warmed saline solution. The distal end of the gastrocnemius muscle was sutured and connected to a wide range force transducer (AD Instruments, Colorado Springs, CO). The resting tension and muscle length were adjusted by a pre-load of 1 g. The left sciatic nerve was exposed through splitting the left biceps femoris muscle along muscle fibers. Maximal electrical tetanus stimulation (10 V, 50 Hz, 0.1-ms pulse, 5-s duration) was produced by a bipolar platinum electrode placed on the distal cut end of the sciatic nerve to induce tetanic contractile force of the gastrocnemius muscle.

After the recording of the skeletal muscle contractile force, a specific muscle Na^+^ channel blocker, *µ*-conotoxin GIIIB (4 μM, 200 μL), was locally injected into the gastrocnemius muscle to inhibit the action potential initiation and propagation, which avoid the influence of skeletal muscle contraction in the EPP recording. A glass microelectrode filled with 3 M KCl (5–15 MΩ pipette resistance) was slowly inserted into the gastrocnemius muscle fiber and connected with an intracellular preamplifier (IX1; Dagan Corporation, Minneapolis, MN, United States) for the EPP recording. The proximity of the electrode to an endplate was determined by the presence of mEPPs. EPPs in 6-8 sites of the gastrocnemius muscle in each mouse were recorded by intracellular recording technique under the electrical stimulation of the exposed sciatic nerve (10 V, 50 Hz, 0.1 ms).

Sciatic nerve stimulation-evoked muscle contractile force and EPPs were digitized by PowerLab 8/30 Data Acquisition System with LabChart 7 (AD Instruments, Colorado Springs, CO, United States), and stored in computer for analyzing the amplitude of muscle contractile force and EPPs.

### Immunohistochemistry of the neuromuscular junction (Tu et al., 2017; Tu et al., 2020)

Gastrocnemius muscles in all groups of mice were quickly isolated for postfixing with 4% paraformaldehyde for 15 min, and subsequently incubating with 0.1 M glycine for 15 min. To facilitate probe penetrations into NMJs, the gastrocnemius muscle from each mouse was divided into 8-10 small longitudinal segments, and then permeabilized in -20°C methanol for 10 min. After blocking with PBS containing 0.5% Triton (BP151, Thermo Fisher Scientific, Waltham, MA) and 1% BSA (A7888, Sigma, St. Louis, MO) for 1 h, small longitudinal segments of the gastrocnemius muscle were incubated overnight at 4°C in a cocktail of primary antibodies, including mouse anti-neurofilament 200 (NF-200, N0142, Sigma-Aldrich, St. Louis, MO) and rabbit anti-synaptophysin (MA5-16402, Thermo Fisher Scientific, Waltham, MA) antibodies, for axon and nerve terminal labeling. Then muscle segments were incubated overnight at 4°C with Alexa Fluor® 594 labeled donkey anti-mouse (A21203, Thermo Fisher Scientific, Waltham, MA) and anti-rabbit (A21207, Thermo Fisher Scientific, Waltham, MA) IgGs, and Alexa Fluor® 488 labeled α-bungarotoxin (α-BTX, B13422, Thermo Fisher Scientific, Waltham, MA). As molecular markers of nerve fibers, nerve terminals, and nicotinic acetylcholine receptors (nAChRs), the specificity of these primary antibodies has been validated in previous published studies (Berg et al., 1972; Mu et al., 2017; Orekhova et al., 2022). Finally, images of muscle segments mounted on glass slides were captured using a laser scanning confocal microscope (Zeiss LSM 800) to analyze immunohistochemically labeled NMJs, including motor nerve terminals and nAChR clusters.

In each muscle segment, 5 different regions were selected to obtain Z-stack images of the NMJ. All analyses were done by ImageJ software (NIH Image) on en-face NMJs. In the NMJ, the percentage of motor nerve innervation and motor nerve occupancy were quantified by measurements of nerve terminals labeled with NF-200 and synaptophysin and nAChRs labeled with α-BTX. Motor nerve occupancy was used to describe the overlap between presynaptic nerve terminals and postsynaptic nAChR clusters and calculated as a percentage of presynaptic nerve terminal area vs. postsynaptic nAChR cluster area. The endplate with or without labelling of neurofilament and synaptophysin was defined as an innervated or denervated endplate, respectively. The nAChR areas in NMJs labeled with α-BTX were used to calculate the whole area per nAChR cluster and the number of discrete fragments per nAChR cluster. A fragmented nAChR cluster was defined when the number of discrete fragments per nAChR cluster ≥ 5.

### Histological evaluation of skeletal muscle alteration

In one experiment, gastrocnemius muscles in all groups of mice were successively fixed in Methacarn solution (300 ml methanol, 150 ml chloroform, and 50 ml acetic acid) for 48 h, and 60% ethanol for 72 h. The fixed muscles were embedded into paraffin wax after routine processing, and then cut into 4-μm-thick longitudinal sections. After deparaffinization, sections were stained with Masson’s trichrome (MT, HT15-KT, Sigma-Aldrich, St. Louis, MO). Stained sections were scanned by Ventana iScan HT scanner (Roche, Switzerland) and adipose area and fibrosis area were measured with ImageJ. The ratio of adipose area or fibrosis area to total section area was calculated.

In another experiment, gastrocnemius muscles in all groups of mice were postfixed with 4% paraformaldehyde for 12 h, and then soaked in 30% sucrose for 12 h at 4°C for cryostat procedure. The muscles were cut into 10-µm-thick cross-sections in a freezing cryostat at -20°C. Tissue sections were processed with standard immunocytochemical staining procedure. Briefly, tissue sections were permeabilized with 0.3% Triton X-100 (BP151, Thermo Fisher Scientific, Waltham, MA) in PBS at room temperature for 20 min. Tissue sections were successively incubated with 10% normal donkey serum (017-000-021, Jackson Immunoresearch Labs Inc.), rabbit anti-dystrophin antibody (ab15277, abcam, Cambridge, MA). After washing with phosphate buffered salts (PBS, 1760420, MP Biomedicals, OH), the sections were incubated with donkey anti-rabbit IgG labelled with Alexa Fluor 594 (A21207, Thermo Fisher Scientific, Waltham, MA), and DAPI (D9542 Sigma-Aldrich, MO, United States). As the molecular marker of skeletal muscle membrane, the specificity of anti-dystrophin antibody was validated by Farea’s study (Farea et al., 2020). The tissue immunofluorescent images were obtained using a Zeiss Observer Z1 microscope (Carl Zeiss AG, Oberkochen, Germany) with a digital camera (Axiocam 503 Mono), and the muscle fiber cross section area was measured with ImageJ.

### Statistical analysis

All data are presented as means ± SEM. SigmaStat 12 was used for data analyses. A two-way ANOVA with post hoc Bonferroni test was used to determine statistical significance for multi-group comparison. Normal distribution of data was confirmed with the Kolmogorov-Smirnov test and equal variance with Levene’s test. Statistical significance was accepted when *p* < 0.05.

## Results

### Ischemia-induced histological alterations in gastrocnemius muscles

**FIGURE 1:**
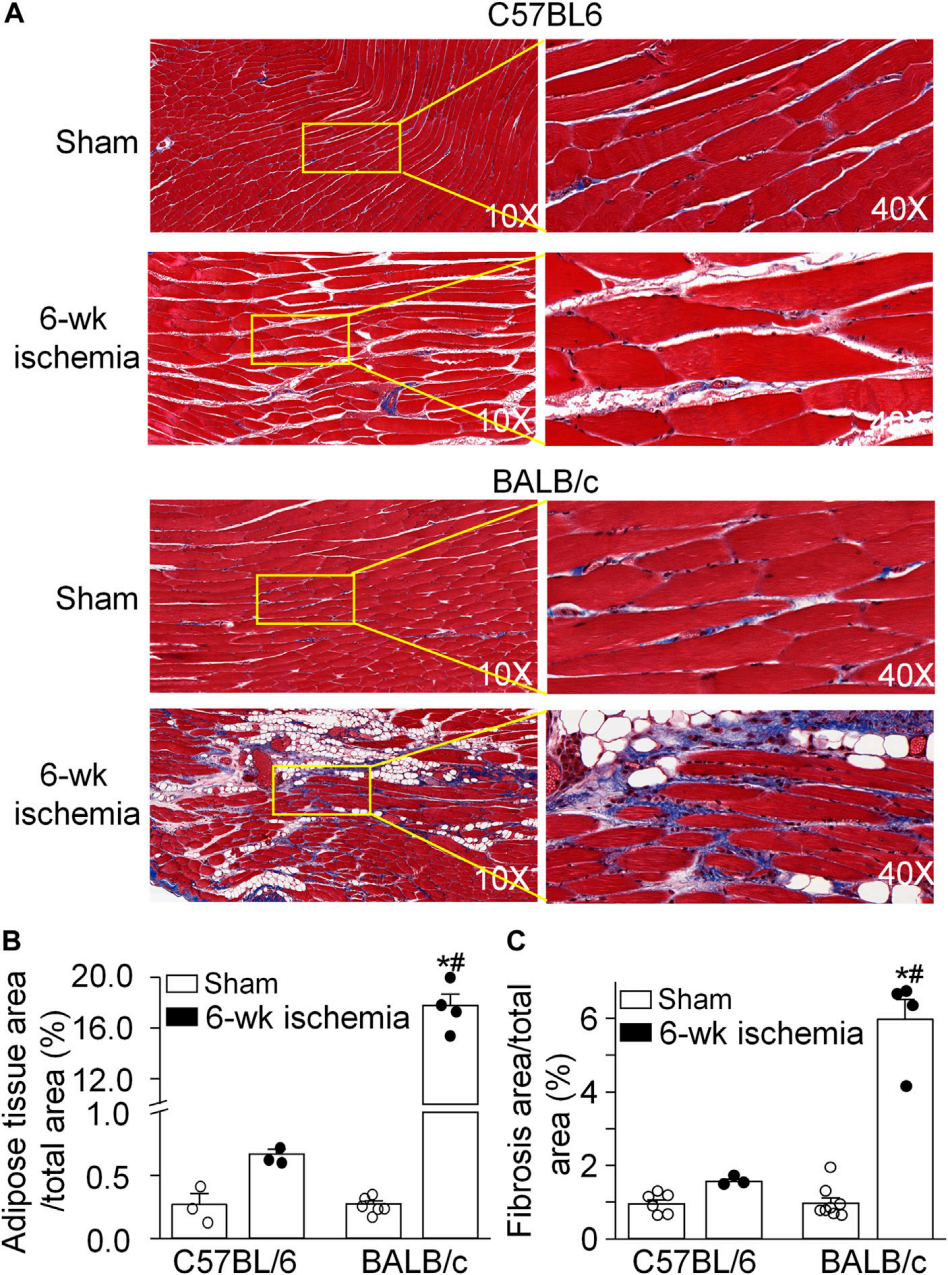
Pathological alterations of gastrocnemius muscl*es* induced by 6 weeks of the femoral artery ligation (Isch) in C57BL/6 and BALB/c mice. **(A)**, representative histological images of the longitudinal muscle section with Masson’s trichrome staining. **(B)** and **(C)**, percentages of adipose tissue and fibrosis area in the gastrocnemius muscle. Data are mean ± SEM, *n* = 6, 3, 4, and 4 mice in C57BL/6 sham, C57BL/6 Isch, BALB/c sham, and BALB/c Isch group, respectively. *p < 0.05 vs. C57BL/6 sham or BALB/c sham, respectively; ^#^ *p* < 0.05 vs. C57BL/6 Isch.

The area of adipose and fibrotic tissue was calculated in the MT stained muscle longitudinal section (Figure 1). There were very few adipose tissue and fibrosis in the gastrocnemius muscle from C57BL/6 sham (0.27 ± 0.09% and 0.95 ± 0.10%) and BALB/c sham mice (0.27 ± 0.03% and 0.97 ± 0.15%) (*p* > 0.05 between C57BL/6 and BALB/c sham mice). After 6 weeks of the femoral artery ligation, the adipose tissue and fibrosis in the gastrocnemius muscle showed a little increase in C57BL/6 mice (0.67 ± 0.04% and 1.56 ± 0.06%, *p* > 0.05, compared to C57BL/6 sham mice). However, a significant increase in the area of adipose tissue (17.77 ± 0.87%) and fibrosis (5.98 ± 0.53%) was observed in the gastrocnemius muscle from BALB/c Isch mice (*p* < 0.05, compared to BALB/c sham and C57BL/6 Isch mice).

**FIGURE 2:**
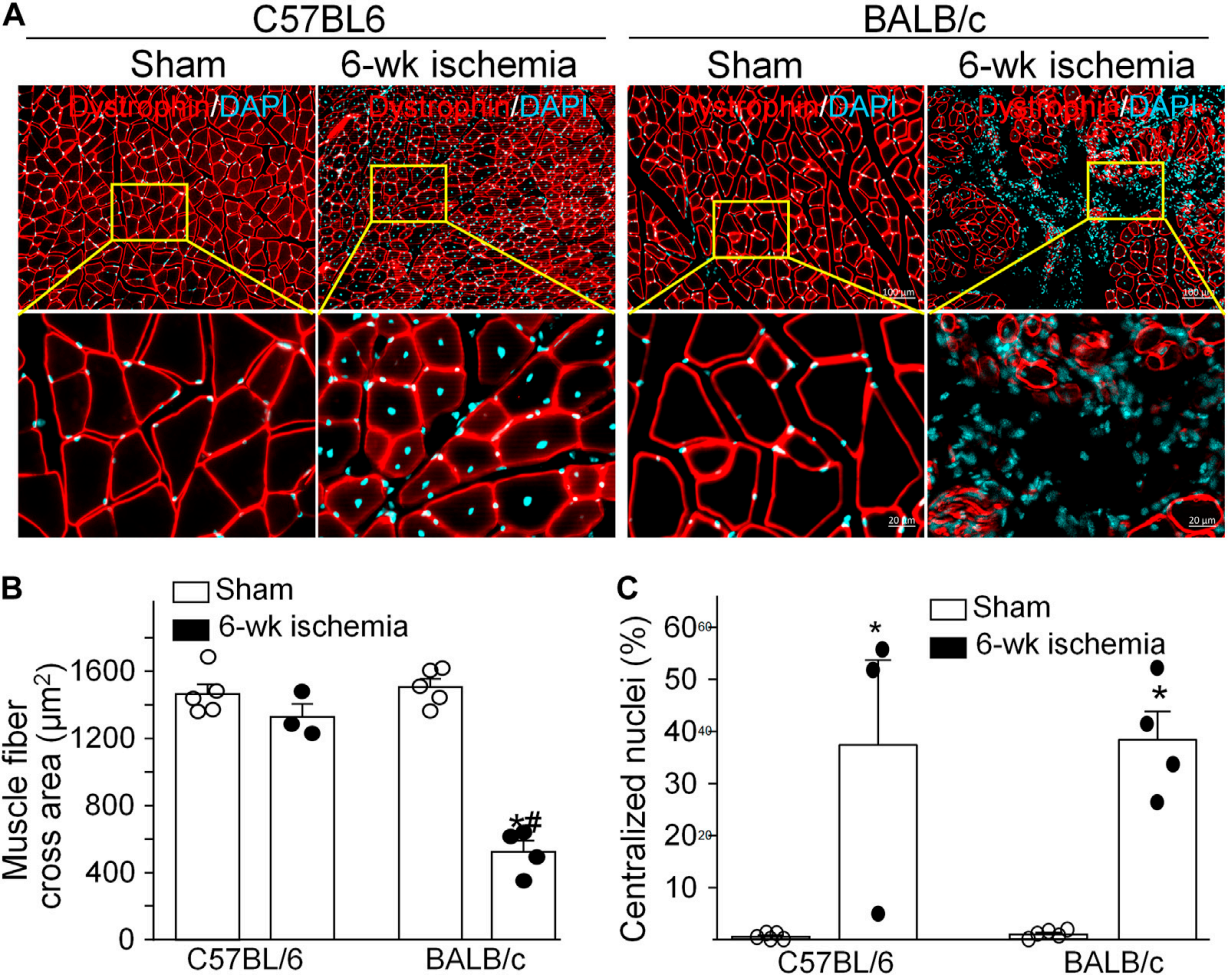
Structural changes of gastrocnemius muscles induced by 6 weeks of the femoral artery ligation (Isch) in C57BL/6 and BALB/c mice. **(A)**, representative immunofluorescence images of cross muscle section with dystrophin staining. **(B)** and **(C)**, summary data for cross sectional area of muscle fibers and the percentage of myofibers with centralized nuclei in gastrocnemius muscles. Data are mean ± SEM, *n* = 5, 3, 5, and 4 mice in C57BL/6 sham, C57BL/6 Isch, BALB/c sham, and BALB/c Isch group, respectively. *p < 0.05 vs. C57BL/6 sham or BALB/c sham, respectively; ^#^ *p* < 0.05 vs. C57BL/6 Isch.

**FIGURE 3:**
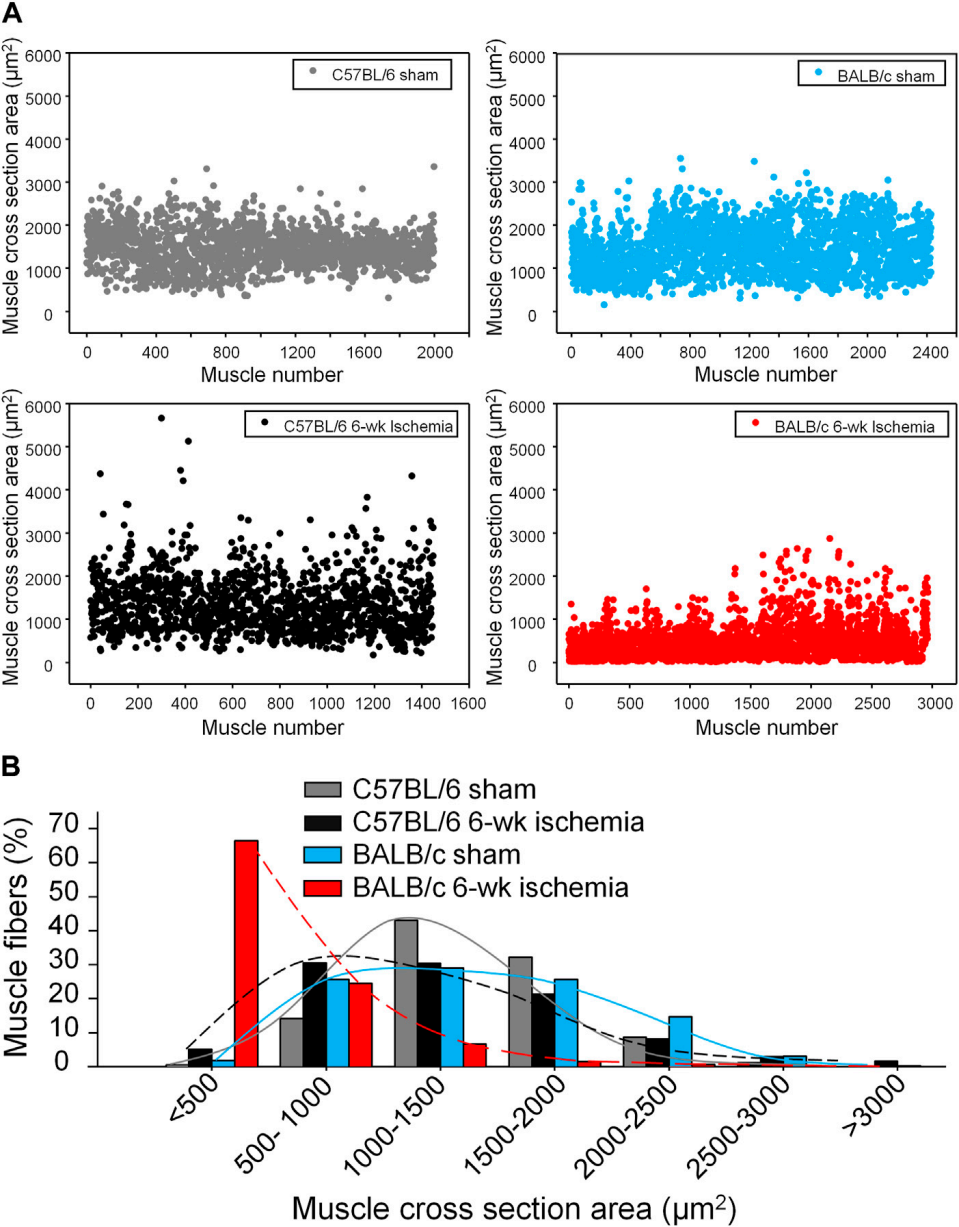
The distribution of myofiber sizes in all experimental groups. **(A)**, dot plots for the myofiber number in each size range. **(B)**, the percentage of the myofiber number in each size range to total number of myofibers.

Structural alterations in the cross section of gastrocnemius muscles were measured with dystrophin immunofluorescence staining (Figure 2). The nuclei of muscles were also labeled by DAPI to calculate the percentage of nucleus-centralized myofibers (Figure 2), because mispositioning of myonuclei is often found in pathological conditions (Folker and Baylies 2013). There was the similar myofiber size with few nucleus-centralized myofibers in C57BL/6 (1,463.67 ± 58.94 µm^2^ and 0.54 ± 0.26%, respectively) and BALB/c sham mice (1,505.30 ± 48.45 µm^2^ and 1.02 ± 0.33%, respectively, *p* > 0.05, Figures 2A–C). After 6 weeks of the femoral artery ligation, the area of myofiber cross section was markedly reduced in BALB/c Isch mice (524.25 ± 66.28 µm^2^, *p* < 0.05, compared to BALB/c sham and C57BL/6 Isch mice) but not in C57BL/6 Isch mice (1,328.28 ± 76.31 µm^2^, *p* > 0.05, compared to C57BL/6 sham mice). Additionally, the nucleus-centralized myofibers were increased in both C57BL/6 Isch (37.42 ± 16.31%) and BALB/c Isch mice (38.36 ± 5.52%) (*p* < 0.05, compared to C57BL/6 and BALB/c sham mice, respectively). Furthermore, the distribution of myofiber cross-section area was significantly changed in BALB/c mice as shown in Figure 3. The myofiber cross-section area was larger than 400 μm^2^ in almost all of myofibers from C57BL/6 (99.7%) and BALB/c (99.51%) sham mice. Six-weeks of the femoral artery ligation non-significantly changed the distribution of myofiber cross-section area in C57BL/6 mice (C57BL/6 Isch group), whereas it caused over 50% of myofibers (55.92%) with cross-section area less than 400 μm^2^ in BALB/c mice (BALB/c Isch group) (Figure 3). These data demonstrated that the femoral artery ligation-induced ischemia significantly increased muscle adipose accumulation and fibrosis with reduction of myofiber cross-section area in BALB/c mice but not in C57BL/6 mice.

### Ischemia-induced functional alterations in gastrocnemius muscles

**FIGURE 4:**
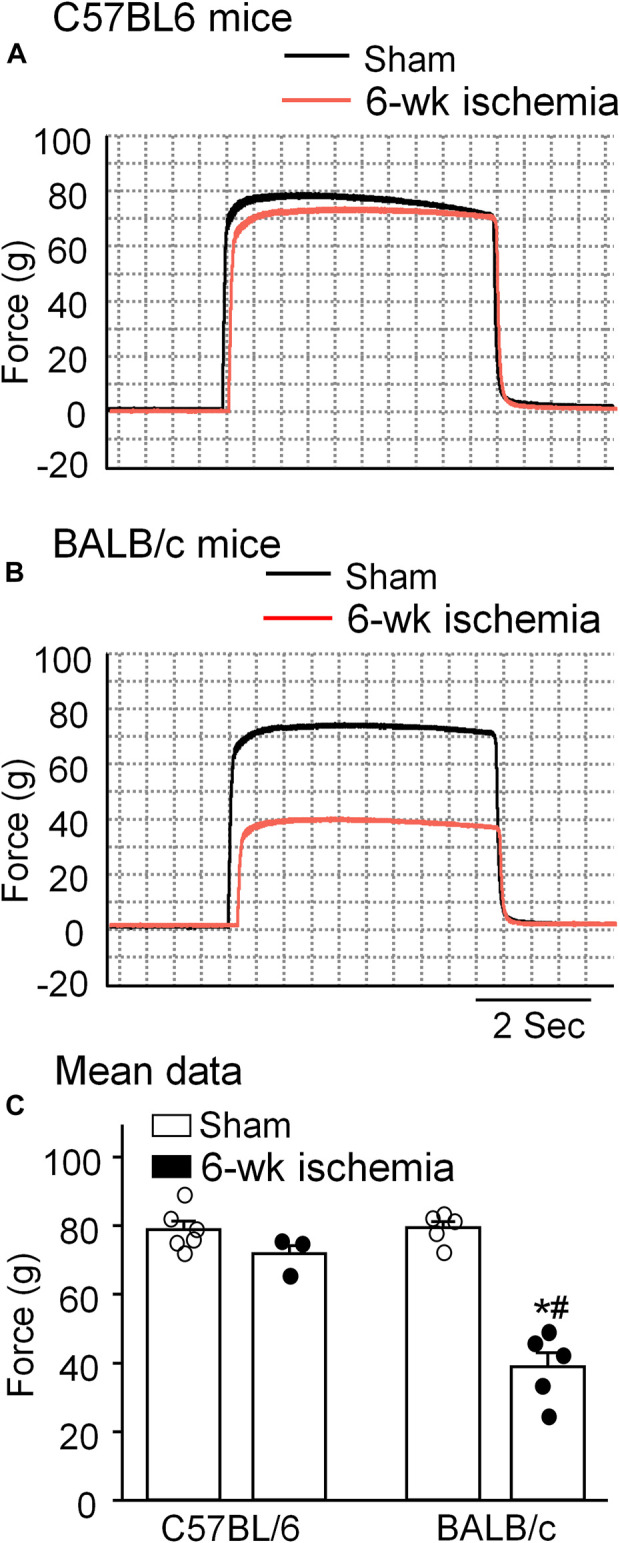
Representative **(A,B)** and summary **(C)** data for *in situ* sciatic nerve-stimulated gastrocnemius muscle tetanic contraction in all experimental groups. Data are mean ± SEM, *n* = 6, 3, 5, and 5 mice in C57BL/6 sham, C57BL/6 Isch, BALB/c sham, and BALB/c Isch group, respectively. *p < 0.05 vs. C57BL/6 sham or BALB/c sham, respectively; ^#^ *p* < 0.05 vs. C57BL/6 Isch.

Skeletal muscle function was evaluated by sciatic nerve stimulation-induced gastrocnemius muscle contraction. The force of gastrocnemius muscle contraction was 78.83 ± 2.47 g in C57BL/6 sham mice and 79.36 ± 1.84 g in BALB/c sham mice, respectively (*p* > 0.05, Figure 4). Six-week ischemia induced by the femoral artery ligation didn’t significantly change the skeletal muscle contraction in C57BL/6 mice (71.87 ± 2.29 g for C57BL/6 Isch mice, *p* > 0.05, compared to C57BL/6 sham mice), whereas it markedly weakened skeletal muscle contraction in BALB/c mice (38.93 ± 4.09 g for BABL/c Isch mice, *p* < 0.05, compared to BALB/c sham and C57BL/c Isch mice) (Figure 4).

### Ischemia-induced morphological changes in neuromuscular junctions

**FIGURE 5:**
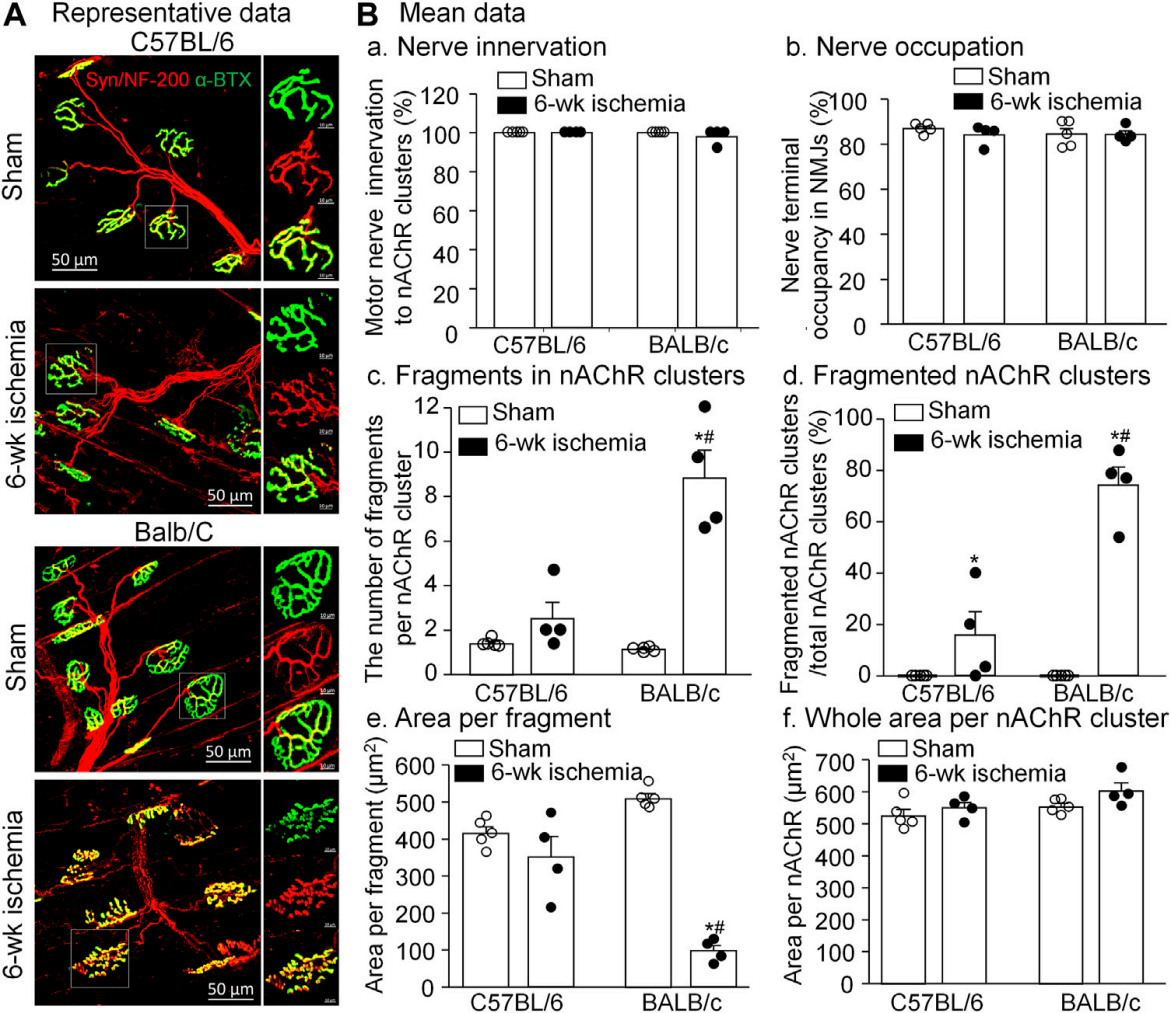
Morphological changes of neuromuscular junctions (NMJs) in gastrocnemius muscles induced by 6 weeks of the femoral artery ligation (Isch) in C57BL/6 and BALB/c mice. **(A)**, representative immunofluorescence images of NMJs. Motor nerve axons and terminals (red color) were labeled with neurofilament 200 (NF-200) and synaptophysin (Syn), and nicotinic acetylcholine receptors (nAChRs, green color) were labeled with Fluor® 488-α-bungarotoxin (α-BTX). Right insets in all groups illustrate amplified photomicrographs for one NMJ. **(B)**, summary data for some parameters of motor nerve terminals and nAChR clusters. Data are mean ± SEM, *n* = 5, 4, 5, and 4 mice in C57BL/6 sham, C57BL/6 Isch, BALB/c sham, and BALB/c Isch group, respectively. *p < 0.05 vs. C57BL/6 sham or BALB/c sham, respectively; ^#^ *p* < 0.05 vs. C57BL/6 Isch.

As the specific synapse in the skeletal muscle, the NMJ transmits the signal from motor neurons to the skeletal muscle for muscle contraction. To observe the NMJ morphology, two major components of NMJs, motor nerve terminals and nAChR clusters, were labeled by neurofilament 200/synaptophysin and α-BTX, respectively (Figure 5A). Normally, the structure of pretzel-like nAChR clusters remained intact, and motor nerve terminals overlapped with nAChR clusters perfectly for their innervation (both C57BL/6 and BALB/c sham in Figure 5A). After 6-weeks ischemia induced by the femoral artery ligation, motor nerve innervation to nAChR clusters and motor nerve terminal occupancy in NMJs in the gastrocnemius muscles did not change in C57BL/6 and BALB/c mice (Figures 5B-a and -b, *p* > 0.05 sham vs. 6-weeks ischemia groups). However, 6-weeks ischemia induced by the femoral artery ligation resulted in the nAChR damage in C57BL/6 and BALB/c mice, demonstrated by the increased number of fragments per nAChR cluster, percentage of fragmented nAChR clusters in total nAChR clusters, and reduced area per fragment of the nAChR cluster (Figures 5B-c, -d, and -e). Importantly, the nAChR damage was more severe in BALB/c mice than that in C57BL/6 mice (Figures 5B-c, -d, and -e).

### Ischemia-induced functional changes in neuromuscular junctions

**FIGURE 6:**
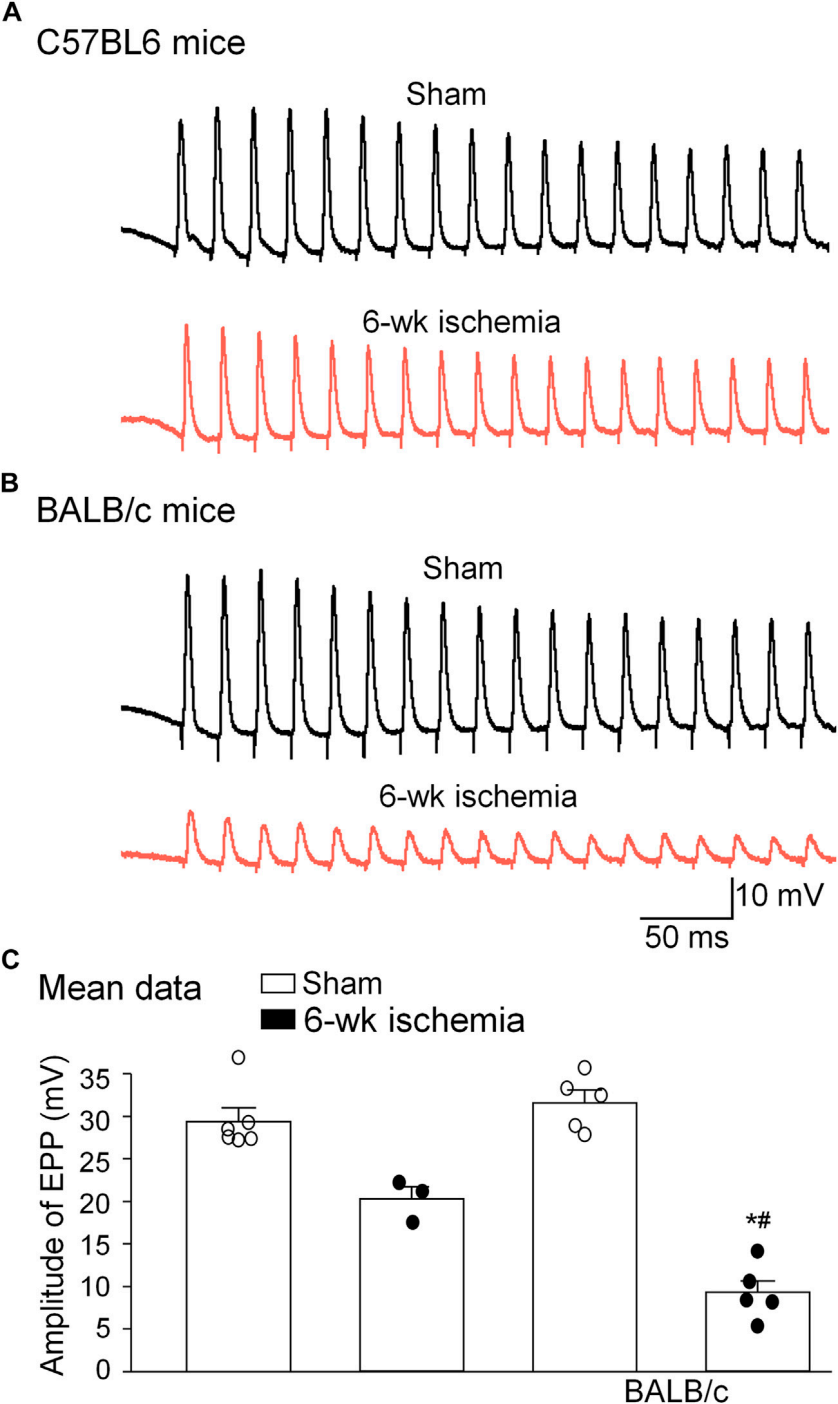
Representative **(A,B)** and summary **(C)** data for sciatic nerve-stimulated endplate potentials (EPPs) in gastrocnemius muscles from all experimental groups. Data are mean ± SEM, *n* = 6, 3, 5, and 5 mice in C57BL/6 sham, C57BL/6 Isch, BALB/c sham, and BALB/c Isch group, respectively. *p < 0.05 vs. C57BL/6 sham or BALB/c sham, respectively; ^#^ *p* < 0.05 vs. C57BL/6 Isch.

As index of the NMJ function, sciatic nerve-stimulated EPPs in the gastrocnemius muscle were recorded (Figure 6). In sham mice, sciatic nerve stimulation evoked regular, steady EPPs (29.5 ± 1.5 mV for C57BL/6 mice and 31.7 ± 1.5 mV for BALB/c mice). After the femoral artery ligation, the amplitude of EPPs was significantly decreased in both C57BL/6 (20.28 ± 1.42 mV) and BALB/c mice (8.92 ± 1.57 mV) (*p* < 0.05 vs. sham groups, respectively), especially more decrease in BALB/c Isch mice (*p* < 0.05 vs. C57BL/6 Isch mice) (Figure 6C).

## Discussion

In the present study, we used a mouse model subjected the femoral artery ligation to simulate critical limb ischemia (CLI), a unique manifestation of PAD and compared the femoral artery ligation-induced morphological and functional changes of the skeletal muscle between C57BL/6 and BALB/c mice. In BALB/c mice, 6 weeks of the femoral artery ligation increased muscle adipose accumulation and fibrosis, resulted in reduction of myofiber cross-section area and nAChR cluster fragmentation, and impaired NMJ transmission and skeletal muscle contraction. However, 6 weeks of the femoral artery ligation only induced mild nAChR cluster fragmentation and slight decrease of EPP amplitudes without muscle adipose accumulation and impairment of skeletal muscle contraction in C57BL/6 mice. These morphological and functional changes in the gastrocnemius muscle from BALB/c mice reproduced pathological symptoms of the skeletal muscle from PAD patients.

Data from computed tomographic imaging demonstrated that PAD patients have smaller calf muscle area with accumulated adipose tissue, compared to the individuals without PAD (Regensteiner et al., 1993; McDermott et al., 2007; Garg et al., 2011; McDermott et al., 2012). Histological studies have also documented a number of abnormalities in calf muscles from PAD patients, including the presence of degenerating and regenerating fibers, reduced skeletal muscle area, connective tissue proliferation and fibrosis, fat accumulation (Hedberg, Angquist, and Sjostrom 1988; Pipinos et al., 2006; Pipinos et al., 2008; Koutakis et al., 2015; McDermott et al., 2020). These adverse calf muscle characteristics are predictors of functional decline and all-cause mortality in this population (McDermott et al., 2009; McDermott et al., 2012). Reversing pathophysiological alterations in calf muscles has been served as the potential therapeutic target to prevent mobility loss in PAD patients (McDermott et al., 2009). Similar to the histopathological changes and functional decline in PAD patients, 6 weeks of the femoral artery ligation in BALB/c mice but not C57BL/6 mice caused muscle adipose accumulation and fibrosis (Figure 1), small cross-sectional area of gastrocnemius myofibers (Figures 2, 3), and weakness of skeletal muscle contraction (Figure 4).

The NMJ plays a fundamental role in signal transmission from lower motor neurons to the skeletal muscle for generation of skeletal muscle contraction. Jones et al. reported that human NMJs are remarkably stable across the entire adult lifespan, showing no signs of age-related degeneration or remodeling (Jones et al., 2017), but another data showed that AChR clusters were fragmented with aging and cardiovascular diseases (Oda 1984), while there are few data on NMJ remodeling in PAD patients. It is reported that denervation and reinnervation of the motor nerve terminals in the NMJ occurred in PAD patients (Regensteiner et al., 1993) and animal models (Mohiuddin et al., 2019). In our present study, although all endplates were innervated after 6 weeks of the femoral artery ligation in both C57BL/6 and BALB/c mice, the structure of nAChR clusters and neuron-muscle transmission (EPPs) were seriously impaired in BALB/c mice and slightly damaged in C57BL/6 mice (Figures 4, 5). These results suggest that fragmentation of nAChR clusters might be involved in EPP suppression and skeletal muscle contractile dysfunction in PAD. However, we cannot rule out functional changes of the motor nerve terminals after 6 weeks of the femoral artery ligation, because we did not measure the acetylcholine release from the motor nerve terminals in our present study.

Normally, nuclei in myofibers are distributed at the periphery of skeletal muscle cells. In most diseases, centralized nuclei in myofibers are caused by muscle wasting, a constant state of muscle degeneration/regeneration (Roman and Gomes 2018). In general, the nuclei positioned in the center of myofibers are a sign of muscle repair (Folker and Baylies 2013; Cadot, Gache, and Gomes 2015). However, nuclear centralization in myofibers also appeared in PAD patients with skeletal muscle degeneration (Vono et al., 2016). Disruption of normal nuclear positioning results in hindered skeletal muscle contraction (Folker and Baylies 2013; Roman and Gomes 2018). Our current study found that the femoral artery ligation significantly increased centralization of muscle fiber nuclei in both C57BL/6 and BALB/c mice (Figure 2C). Based on the above data, the skeletal muscle suffered degeneration and regeneration after the femoral artery occlusion in both C57BL/6 and BALB/c mice, though C57BL/6 mice showed faster muscle repairment than BALB/c mice.

Both C57BL/6 (Pipinos et al., 2008; Mohiuddin et al., 2019) and BALB/c (Roseguini et al., 2015; Goldberg et al., 2019) mice were used to explore the femoral artery ligation-induced skeletal muscle structural and functional alterations. Like a previous study (Schmidt et al., 2017), our current study found that C57BL/6 mice displayed faster recovery of myofiber structure and function, compared to BALB/c mice. It is thought that genetic background of mouse strain influences the outcome (Dokun et al., 2008; Aref, de Vries, and Quax 2019). By genetic comparison between C57BL/6 and BALB/c mice, Okeke and Dokun (Okeke and Dokun 2018) summarized that polymorphism in genes can influence perfusion recovery and extent of limb necrosis following induction of hindlimb ischemia in different mouse strains. As genetic modifiers of endothelial cell proliferation, survival, and angiogenesis, microRNA-93 (Hazarika et al., 2013), ADAM12, and IL-21Rα (Okeke and Dokun 2018) are more highly upregulated in C57BL/6 mice, compared to BALB/c mice after the femoral artery ligation. Some studies have demonstrated that C57BL/6 mice display a high degree of neovascularization and fast recovery of limb perfusion after hind limb ischemia in comparison to a slow recovery of BALB/c mice (Helisch et al., 2006; Chalothorn and Faber 2010). Furthermore, muscle cells from BALB/c mice are more susceptible to ischemia than that from C57BL/6 mice (McClung et al., 2012). Thus, the C57BL/6 strain typically showed favorable perfusion recovery with little or no tissue necrosis, whereas the BALB/c strain typically showed poor perfusion recovery with profound tissue necrosis.

Surgical procedure also affects the vascular perfusion (Shireman and Quinones 2005). Both femoral artery and vein ligation and transection result in severe tissue damage and gangrene in BALB/c mice (Parikh et al., 2018), whereas, mice with ligation of the femoral artery had no sign of gangrene other than mild darkening of the nails in some animals (Goldberg et al., 2019). We also found autoamputation of the leg in BALB/c mice with ligation and transection of the femoral artery (data not shown). In our current study, only the femoral artery was ligated to induce moderate ischemic damage in the skeletal muscle and NMJ in BALB/c mice.

Although the femoral artery ligation in BALB/c mice simulates the pathological and functional alterations of the skeletal muscle in PAD patients, the process of PAD development in this animal model is different from clinical PAD. In PAD patients, arterial narrowing and decrease in the blood flow develop gradually over a time span of months to years. Controversially, femoral artery ligation results in acute blood supply interruption to the whole muscle in the animal model (Krishna, Omer, and Golledge 2016). Although a two-stage limb ischemia model (Krishna et al., 2020) and an animal model with gradual arterial occlusion (Tang et al., 2005) can simulate the process of PAD development, the muscle damage in these animal models can’t stably duplicate the pathological changes in PAD patients. Our data showed that the femoral artery ligation in BALB/c mice reproduced the pathological and functional alterations of the skeletal muscle in PAD patients. In particular, these pathological changes are stably kept for a long period (up to 18 weeks, data not shown). Therefore, although femoral artery ligation in BALB/c mice cannot be used to explore the mechanisms underlying the clinical PAD, it is a useful animal model to develop new therapeutic approaches to improve limb structure and function (including the NMJ and skeletal muscle) and manage the outcome of PAD.

One limitation of the current study is that only male mice are used. We understand clearly that if male and female mice are body weight-matched, the females are older than the males; if they are age-matched, the males are heavier than the females. The difference of both the age and body weight can affect the stability and replication of the femoral artery ligation-induced hindlimb ischemic injury. In addition, sex-based differences in skeletal muscle fiber-type composition, capillary density and function (Haizlip, Harrison, and Leinwand 2015; O'Reilly et al., 2021) also affect the comparison of the femoral artery ligation-induced hindlimb ischemic injury among different mouse strains. For the sake of clarify and focus, therefore, we only investigated different responses of skeletal muscles to femoral artery ligation-induced ischemia in C57BL/6 and BALB/c male mice. As a biological variable in basic and clinical studies, sex-based difference in PAD induction should be addressed in future studies.

## Conclusion

6 weeks of femoral artery ligation induced severe structural and functional impairments of the NMJs and skeletal muscles in BALB/c mice, whereas it only resulted in a mild damage of the NMJs and skeletal muscles in C57BL/6 mice. Compared to C57BL/6 mice, BALB/c mice with femoral artery ligation offers a useful model to explore how to improve limb function and prognosis of PAD.

## Data Availability

The original contributions presented in the study are included in the article/supplementary material, further inquiries can be directed to the corresponding author.
